# Thermal Ablation versus Stereotactic Ablative Body Radiotherapy to Treat Unresectable Colorectal Liver Metastases: A Comparative Analysis from the Prospective Amsterdam CORE Registry

**DOI:** 10.3390/cancers13174303

**Published:** 2021-08-26

**Authors:** Sanne Nieuwenhuizen, Madelon Dijkstra, Robbert S. Puijk, Florentine E. F. Timmer, Irene M. Nota, Jip Opperman, Bente van den Bemd, Bart Geboers, Alette H. Ruarus, Evelien A. C. Schouten, Jan J. J. de Vries, Hester J. Scheffer, Anne M. van Geel, Jan Hein T. M. van Waesberghe, Rutger-Jan Swijnenburg, Kathelijn S. Versteeg, Birgit I. Lissenberg-Witte, M. Petrousjka van den Tol, Cornelis J. A. Haasbeek, Martijn R. Meijerink

**Affiliations:** 1 Department of Radiology and Nuclear Medicine, Amsterdam UMC, Cancer Center Amsterdam, VU University, 1081 HV Amsterdam, The Netherlands; m.dijkstra3@amsterdamumc.nl (M.D.); r.puijk@amsterdamumc.nl (R.S.P.); f.timmer1@amsterdamumc.nl (F.E.F.T.); im.nota@amsterdamumc.nl (I.M.N.); b.vandenbemd@amsterdamumc.nl (B.v.d.B.); b.geboers@amsterdamumc.nl (B.G.); a.ruarus@amsterdamumc.nl (A.H.R.); e.schouten@amsterdamumc.nl (E.A.C.S.); j.devries1@amsterdamumc.nl (J.J.J.d.V.); hj.scheffer@amsterdamumc.nl (H.J.S.); jhtm.vanwaesberghe@amsterdamumc.nl (J.H.T.M.v.W.); mr.meijerink@amsterdamumc.nl (M.R.M.); 2 Department of Radiology and Nuclear Medicine, Noordwest Ziekenhuisgroep, 1815 JD Alkmaar, The Netherlands; j.opperman@nwz.nl (J.O.); a.m.van.geel@nwz.nl (A.M.v.G.); 3 Department of Surgery, Amsterdam UMC, Cancer Center Amsterdam, VU University, 1081 HV Amsterdam, The Netherlands; r.j.swijnenburg@amsterdamumc.nl; 4 Department of Medical Oncology, Amsterdam UMC, Cancer Center Amsterdam, VU University, 1081 HV Amsterdam, The Netherlands; k.versteeg@amsterdamumc.nl (K.S.V.); mp.vandentol@amsterdamumc.nl (M.P.v.d.T.); 5 Department of Epidemiology and Data Science, Amsterdam UMC, Cancer Center Amsterdam, VU University, 1081 HV Amsterdam, The Netherlands; b.lissenberg@amsterdamumc.nl; 6 Department of Radiation Oncology, Amsterdam UMC, Cancer Center Amsterdam, VU University, 1081 HV Amsterdam, The Netherlands; cja.haasbeek@amsterdamumc.nl

**Keywords:** colorectal liver metastases (CRLM), thermal ablation, microwave ablation (MWA), radiofrequency ablation (RFA), stereotactic ablative radiotherapy (SABR)

## Abstract

**Simple Summary:**

Approximately 30–50% of colorectal cancer patients will develop colorectal liver metastases (CRLM) in the course of their disease. Though partial hepatectomy is considered the historical gold standard, complete removal of all metastases is only feasible in 20–30% of patients. Thermal ablation and stereotactic ablative radiotherapy (SABR) are techniques to eradicate unresectable CRLM. This AmCORE based study compares the safety, efficacy and survival outcomes of these treatment methods. In this study thermal ablation was superior to SABR with regard to overall survival, local tumor progression-free survival and local control. However, there was a slightly higher risk of serious adverse events after thermal ablation. Further studies are required to assess whether the worse outcomes following SABR were the effect of true differences in ablative treatment or a result of residual confounding.

**Abstract:**

Thermal ablation and stereotactic ablative radiotherapy (SABR) are techniques to eradicate colorectal liver metastases (CRLM). This study compares the safety, efficacy and long-term oncological outcomes of these treatment methods. All prospectively registered patients (AmCORE registry) treated with thermal ablation or SABR alone for unresectable CRLM between 2007 and 2020 were analyzed using multivariate Cox-proportional hazard regression. In total 199 patients were included for analysis: 144 (400 CRLM) thermal ablation; 55 (69 CRLM) SABR. SABR patients were characterized by older age (*p* = 0.006), extrahepatic disease at diagnosis (*p* = 0.004) and larger tumors (*p* < 0.001). Thermal ablation patients were more likely to have synchronous disease, higher clinical risk scores (*p* = 0.030) and higher numbers of CRLMs treated (*p* < 0.001). Mortality was zero and morbidity low in both groups: no serious adverse events were recorded following SABR (*n* = 0/55) and nine (*n* = 9/144 [6.3%]; all CTCAE grade 3) after thermal ablation. SABR was associated with an inferior overall survival (OS) (median OS 53.0 months vs. 27.4 months; HR = 1.29, 95% CI 1.12–1.49; *p* = 0.003), local tumor progression-free survival (LTPFS) per-tumor (HR = 1.24, 95% CI 1.01–1.52; *p* = 0.044) and local control per-patient (HR = 1.57, 95% CI 1.20–2.04; *p* = 0.001) and per-tumor (HR = 1.89, 95% CI 1.44–2.49; *p* < 0.001). In this study thermal ablation was superior to SABR with regard to OS, LTPFS and local control, albeit at the cost of a limited risk of serious adverse events. Further studies are required to assess whether the worse outcomes following SABR were the effect of true differences in ablative treatment or a result of residual confounding.

## 1. Introduction

Colorectal cancer (CRC) ranks third among cancers in terms of incidence and is the second most common cause of cancer-related mortality in the world, accounting for about one in 10 cancer cases and deaths [1]. The liver is the most common site of distant spread. Approximately 15–25% of CRC patients will have colorectal liver metastases (CRLM) at the time of diagnosis of the primary tumor and another 18–25% patients will develop distant metastases within five years [2,3,4]. A multidisciplinary approach to managing CRLM has been pivotal to recent improvements in survival. Though partial hepatectomy is considered the historical gold standard, with five-year survival rates nowadays reaching 40–55% [5,6,7], complete removal of all metastases is only feasible in 20–30% of patients [2,3,8]. For patients with CRLM that are partially or completely considered unresectable, several other local eradicative treatments have emerged.

The most well-known and widely adopted treatments are thermal ablation techniques, such as radiofrequency ablation (RFA) and microwave ablation (MWA), and non-thermal ablative methods, such as stereotactic ablative radiotherapy (SABR) and most recently irreversible electroporation (IRE). Because of its safety profile and good local control rate, (inter)national multidisciplinary guidelines and expert consensus groups generally recommend the use of thermal ablation for small-size unresectable CRLM [9,10,11] and the reservation of SABR for CRLMs that are unsuitable for partial hepatectomy and thermal ablation, either due to a certain anatomical location, i.e., near critical structures like major blood vessels or bile ducts, or due to a poor general health status [12,13]. Furthermore, thermal ablation is restricted by tumor size as efficacy decreases exponentially for tumors >3 cm [14,15,16,17,18,19]. Hypothetically, SABR can overcome some of these limitations as it does not require the placement of needle electrodes or antennas, is presumably less affected by tumor size and by proximity of large blood vessels, and may be less susceptible to operator experience [20].

In the absence of randomized controlled trials or robust retrospective comparative series, claims of superiority of one technique over the other seem unsubstantiated. The aim of this study was to compare the safety, local control rate and long-term oncological outcome of thermal ablation versus SABR for unresectable CRLM by analyzing per-patient, per-procedure and per-tumor data from the prospective Amsterdam colorectal liver metastases registry (AmCORE).

## 2. Materials and Methods

### 2.1. Study Design and Population

AmCORE is a prospectively maintained database that currently contains coded per-patient, per-procedure and per-tumor data of 737 CRLM patients treated with partial hepatectomy, thermal ablation and/or non-thermal ablation techniques in one of the Amsterdam University Medical Centers (location VUmc) from January 2007 onwards. From that registry, all patients with a history of solely thermal ablation(s) or SABR for previously untreated CRLM within a single session were selected, disallowing concomitant resections for additional CRLM within that session or as a first or second stage to that procedure. A history of previous partial hepatectomy (+/− thermal ablations) was allowed, as long as the CRLMs assessed were newly appearing and hence local treatment-naïve. If a patient had both a treatment-site recurrence from that earlier surgical procedure and a new CRLM, the treatment-site recurrence was disregarded in the evaluation. In case of multiple thermal ablation or SABR sessions, only the first ablative procedure, and the (new) metastases treated in that specific procedure, was analyzed. Patients who had simultaneous bowel surgery were excluded to avoid including complications unrelated to the ablative procedure. To update the registry with missing information, the regional hospitals’ electronic patient databases were consulted. To confirm radiology and nuclear medicine reports when pathology confirmation was unavailable, two independent abdominal radiologists (JHvW and IN, who have 15 years and 3 years of board certified experience, respectively) re-assessed the cross-sectional imaging exams of patients. Imaging re-evaluations were performed independently; discrepancies were noted and resolved using consensus. The institutional review board judged that the study was not subject to the Medical Research Involving Human Subjects Act (METc VUmc: 2020.454).

### 2.2. Radiofrequency Ablation, Microwave Ablation and Stereotactic Ablative Radiotherapy

All patients were discussed at a multidisciplinary tumor board, with the routine presence of interventional radiologists, radiation oncologists, hepatopancreaticobiliary surgeons, medical oncologists, diagnostic radiologists, nuclear medicine physicians and pathologists. Partial hepatectomy was considered the standard treatment method. Conforming to (inter)national guidelines, thermal ablation and SABR were considered appropriate for anatomically unresectable CRLM and for patients with poor performance status and/or major comorbidities. In recent years patients with solitary and deep-seated CRLMs, where resection would require major hepatectomy, have been offered thermal ablation or SABR as a fair alternative to partial hepatectomy. Though thermal ablation was considered the standard for unresectable CRLM, direct referral to radiation oncology from an external center, specific patient’s preference, high age, poor performance status, major cardiopulmonary comorbidities or a perivascular, peribiliary or difficult-to-reach anatomical location were relative criteria to opt for SABR over thermal ablation. 

As this study addresses procedures performed over a period of >10 years, both the stereotactic ablative radiotherapy systems and specific dosing and sequencing and the thermal ablation generators, needle electrodes and antenna design, as well as the quality of work-up and follow-up cross-sectional anatomical and molecular imaging, anesthesia techniques and the methods used for real-time image-guidance, needle navigation and ablation confirmation, have undergone substantial technological and methodological developments.

### 2.3. Thermal Ablation

RFA and MWA procedures were preferably performed using a real-time fluoroscopy computed tomography (CT)-guided (+/− ultrasound) percutaneous approach. An ultrasound-guided open or laparoscopic approach was used for (a) potentially resectable CRLM, (b) multiple (>3) CRLM or (c) distancing of certain delicate structures such as intestines if a pneumo- or hydrodissection was unfeasible. Percutaneous thermal ablation procedures were performed under general anesthesia, midazolam sedation or propofol sedation in an interventional oncology treatment room which accommodated an angiography system, CT scanner, ultrasound apparatus and anesthetic facilities. RFA was most often performed using the RF3000 generator with expandable LeVeen electrodes (Boston Scientific, Marlborough, MA, USA), or the RITA system with compatible expandable electrodes (AngioDynamics BV, Amsterdam, The Netherlands) and MWA using the Evident or Emprint (Medtronic-Covidien, Minneapolis, MN, USA) or Solero (AngioDynamics BV, Amsterdam, The Netherlands) generators with compatible antennas. The ablations were performed according to the instructions for use provided by the manufacturer. Overlapping ablations were allowed to treat residual unablated tumor tissue in case of presumed insufficiently ablated margins. As computational techniques to confirm tumor-free margins using image fusion and registration were not available in the earlier years of this assessment, the documented minimum tumor free margins for procedures performed >5 years ago were eyeballed and estimated. After the procedure, patients were admitted to the post-anesthesia care unit to monitor vital parameters and patients remained admitted at the short-stay interventional oncology ward for one night.

### 2.4. Stereotactic Ablative Radiotherapy

Patients were simulated and treated in supine positions according to institutional protocol. The use of gated treatment or breath hold was determined at the discretion of the treating radiation oncologist. SABR was delivered in an image-guided hypofractionated scheme of 60Gy in 3, 5, 8 or 12 fractions, prescribed to 95% of the planning target volume (PTV). Target volumes were delineated by the treating radiation oncologist by means of the available cross-sectional imaging studies. A margin of 3–10 mm was used to delineate the PTV, depending on treatment technique and tumor visibility. Many different irradiation devices from several providers were used over the years. Since 2016 most patients were treated during breath hold using the ViewRay MRIdian system (ViewRay, Oakwood Village, OH, USA). The duodenum, stomach, bowel, liver, gallbladder, kidneys, and spinal cord were contoured as structures to avoid. 

### 2.5. Follow-Up

Recommended follow-up after thermal ablation consisted of three-monthly serum CEA and [18F]-fluoro-2-deoxy-D-glucose (18F-FDG) positron emission tomography (PET)-ceCT scans for the first year of follow-up, changing to six-monthly for the second and third years and then to annually up to five years following the procedure. Follow-up recommendations after SABR were more heterogeneous, but consisted at the very minimum of a six-monthly cross-sectional ceCT scan and serum CEA for the first three years changing to annually up to five years. Contrast enhanced Magnetic Resonance Imaging (ceMRI) including high B-value diffusion-weighted images (DWI) and/or image-guided core biopsies were used as problem solver to confirm or exclude the presence of viable tumor tissue at the treatment site. If histopathological confirmation was not available, local tumor progression (LTP) following thermal ablation and SABR was defined as an unequivocal solid and enlarging, preferably nodular and ring-enhancing, mass at the surface of the ablated tumor and/or a new or enlarging marginal focus of 18F-FDG PET avidity, without signs of inflammation or infection. 

Adverse events, defined as any actual or potential injury related to the ablative procedure, were determined according to the Common Terminology Criteria for Adverse Events 5.0 (CTCAE). Time-to-event endpoints were defined and calculated in accordance with the standardization of short-, mid- and long-term oncological outcome measures in image-guided tumor ablation consensus guidelines. Overall survival (OS) and distant progression-free survival (DPFS) were defined as the times elapsed from the (start of) the ablative treatment to death or to any disease recurrence distant from the ablation site. Local tumor progression-free survival (LTPFS) was defined as the time between (start of) the ablative treatment and LTP per-tumor treated (per-tumor analysis) or per-patient treated (per-patient analysis). For patients alive without LTP at the end of assessment, the date of the last cross-sectional imaging reliably excluding LTP was used as the censoring date. Death without LTP was not considered an event in the LTPFS analysis (it was treated as a competing risk). Local control per-tumor and per-patient is herein defined as the time elapsed between the (start of) the first ablation and the latest date of detection of LTP considered unfeasible for repeat local treatment, including repeat treatments whenever performed; simultaneous discovery of widespread distant disease progression was considered a competing risk. Though potentially correlated, the presence of multiple CRLM in one unique patient was ignored in the per-tumor LTPFS analysis and a per-patient LTPFS analysis was added conforming to recommendations by Puijk et al. [21]. Median follow-up time was calculated for patients alive at the time of analysis.

### 2.6. Statistical Analyses 

Baseline patient, disease, tumor and treatment characteristics were analyzed using the Chi-square test for categorical variables and the independent two-sample *t*-test, ANOVA or Mann–Whitney U test for continuous variables. Characteristics assessed were: age, sex, simplified Charlson’s comorbidity index (CCI < 5, 5–8 or >8), BMI, number and size of metastases, primary tumor location, histopathological T-, N- and M-stage following primary tumor resection, Fong’s clinical risk score (low risk if CRS 0–2; high risk if CRS 3–5; 1 point for baseline CEA level > 200 ng/mL, for node positivity, for >1 CRLM, for largest CRLM > 5 cm and for detection of CRLM within 12-months following primary tumor diagnosis), extrahepatic disease at time of diagnosis of CRLM, history of partial hepatectomy and the use of induction or neo-adjuvant systemic therapy for the CRLM. OS, DPFS and LTPFS were estimated using Kaplan–Meier survival curves, log-rank tests and Cox proportional hazards survival models. Factors with *p* ≤ 0.10 in univariate analysis were entered into the multivariate analysis model to adjust for potential confounders. Statistical analyses were performed using SPSS version 26.0 and R version 4.0.4.

## 3. Results

### 3.1. Baseline and Treatment Characteristics

**Figure 1:**
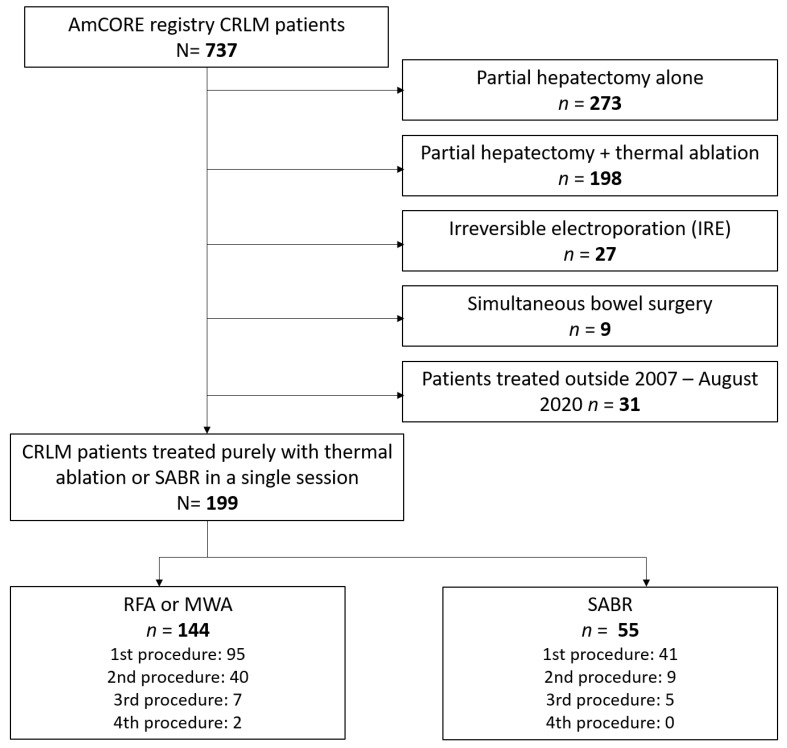
Flowchart of selected patients from the prospective Amsterdam Colorectal Liver Metastases Registry (AmCORE). CRLM = colorectal liver metastases, RFA = radiofrequency ablation, MWA = microwave ablation, SABR = stereotactic ablative radiotherapy.

Between January 2007 and August 2020, 737 registered patients underwent local treatment for CRLM (Figure 1). Of these, 536 patients were excluded because they underwent partial hepatectomy alone (*n* = 273), thermal ablation simultaneous with partial hepatectomy (*n* = 198), with bowel surgery (*n* = 9) or with IRE of at least one of the CRLM within the procedure assessed (*n* = 27). The remaining 199 patients (137 male/62 female; mean age 67.3 ± 10.5 years) were included for full analysis: 144 patients with 400 CRLM underwent thermal ablation and 55 patients with 69 CRLM underwent SABR.

Median follow-up time was 29.3 months for the whole cohort, 29.6 months (range 3.8–168.1) for thermal ablation and 27.6 months (range 11.8–91.3) for SABR. The thermal ablation approach was percutaneous in 55.6% (*n* = 80; 152 CRLM) and open in 44.4% (*n* = 64; 247 CRLM); 63 patients received treatment with RFA (*n* = 63/144; 43.8%), all prior to 2017, and 81 patients (*n* = 81/144; 56.3%) had MWA. In the SABR group 31 patients (*n* = 31/55; 56.4%) were treated with conventional SABR and 24 patients (*n* = 24/55; 43.6%) with MR-guided SABR, all after 2014. Table 1 shows the clinicopathological characteristics of included patients and their distribution over the two treatment groups.

**Figure 2:**
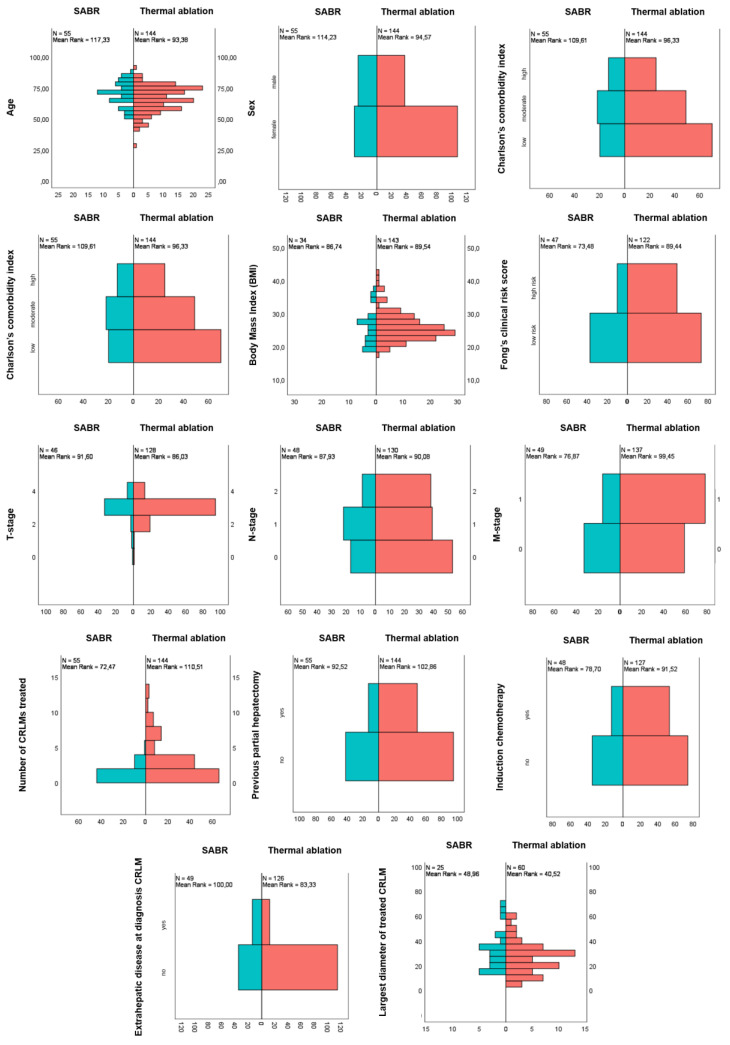
Patients treated with SABR (blue) were older, had larger tumors and higher rates of extrahepatic disease. Patients treated with thermal ablation (red) had higher numbers of CRLMs, higher Fong’s clinical risk scores and higher rates of synchronous disease. All other variables were evenly distributed.

The following potentially confounding parameters were evenly distributed over both treatment groups: CCI, previous chemotherapy regimens and the rate of patients with a history of earlier treatments for CRLM (Figure 2). There appeared to be a trend towards higher CCI scores in the SABR group, however this was not statistically significant. Patients in the SABR group were more often characterized by female sex (*p* = 0.005), higher age (*p* = 0.006), higher rates of extrahepatic disease at diagnosis of CRLM (*p* = 0.004) and larger tumors (*p* < 0.001). Patients treated with thermal ablation had higher Fong clinical risk scores (*p* = 0.030), higher rates of synchronous disease (*p =* 0.005) and higher number of tumors treated (*p* = 0.0004).

### 3.2. Adverse Events and Hospital Stay

Ninety-day mortality was 0% in both groups. Nine patients (*n =* 9/144; 6.3%) developed serious adverse events, all following thermal ablation (all CTCAE grade 3): bile leakage requiring percutaneous drainage (*n =* 2; 1.4%), intrahepatic abscess requiring percutaneous drainage (*n =* 2/144; 1.4%), hepatic hemorrhage requiring coil embolization (*n =* 2/144; 1.4%), bacteremia (*n =* 1/144; 0.7%), ileus (*n =* 1/144; 0.7%) and intraoperative stomach perforation (*n =* 1/144; 0.7%). Lower grade adverse events reported were pain requiring pain medication (*n =* 2/144; 1.4%), abscess requiring antibiotics (*n =* 1/144; 0.7%), hematoma (*n =* 1/144; 0.7%) and pneumothorax requiring no intervention (*n =* 4/144; 2.8%), which amounts to a total of eight CTCAE grade 1 or 2 adverse events (*n =* 8/144; 5.6%). Following SABR, no serious adverse events were registered, and the reported lower-grade adverse events were nausea requiring anti-emetics (*n =* 5/55; 9.1%), pain requiring additional pain medication (*n =* 2/55; 3.6%), rib insufficiency fracture (*n =* 1/55; 1.8%), dysphagia (*n =* 1/55; 1.8%) and fatigue (*n =* 9/55; 16.4%), which totals seventeen CTCAE grade 1 or 2 adverse events (*n =* 17/55; 30.9%). Though more adverse events were reported with SABR (30.9% vs. 11.8%; *p =* 0.0014), thermal ablation was associated with more serious complications that required an intervention (0% vs. 6.3%; *p =* 0.058).

Median hospital stay for thermal ablation was one day (range 1–35 days): one day for percutaneous ablations and five days for open ablations, with one reported hospital readmission. SABR was always performed in the outpatient clinic and patients underwent a median of eight (range 3–12) radiation cycles (not including the planning CT hospital visit) within a median of 16 days (range 5–24 days).

### 3.3. Overall Survival and Distant Progression-Free Survival

Baseline characteristics associated with an inferior OS in univariate assessment were major comorbidities (HR 1.92; 95% CI: 1.16–3.17; *p =* 0.03), node-positive primary tumor (HR 1.51; 95% CI: 0.96–2.38; *p =* 0.07), history of liver resection (HR 0.64; 95% CI: 0.41–1.02; *p =* 0.06), diameter of largest CRLM treated >3 cm (HR 1.81; 95% CI: 1.21–2.70; *p =* 0.004) and SABR treatment (HR 1.29; 95% CI: 1.12–1.49; *p =* 0.003) (see Table 2). 

**Figure 3:**
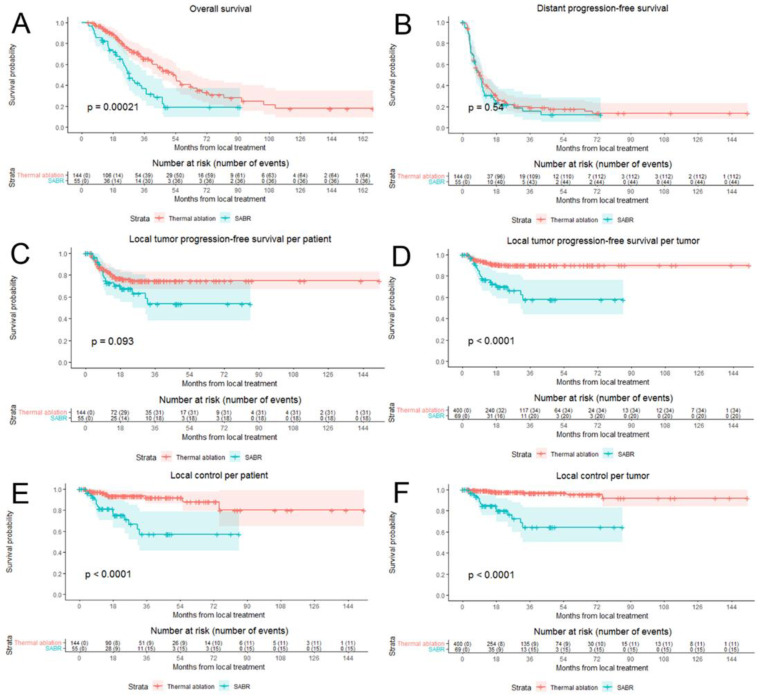
Survival curves following thermal ablation (red) and stereotactic ablative radiotherapy (SABR) (blue) for unresectable colorectal liver metastases: overall survival (**A**), distant progression-free survival (**B**), local tumor progression free survival, per-patient and per-tumor (**C**,**D**) and local control allowing repeat treatment, per-patient and per-tumor (**E**,**F**).

**Figure 4:**
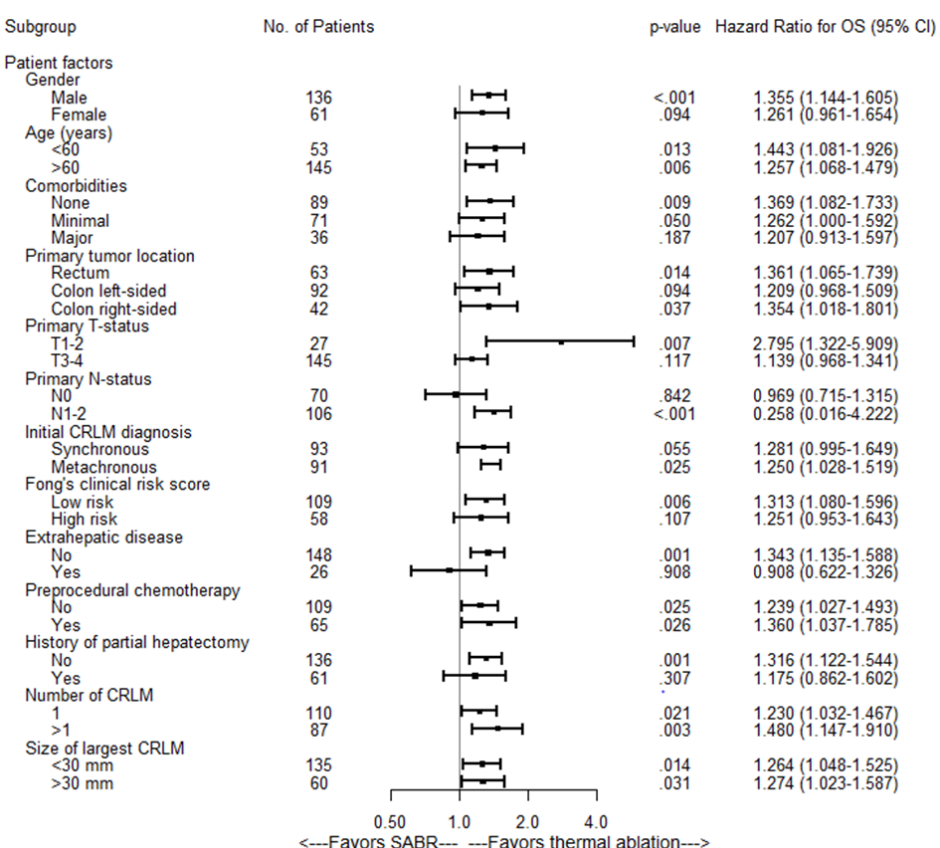
Univariate Subgroup Cox Regression Analyses of SABR versus thermal ablation associated with OS.

After correcting for potential confounders in multivariate analysis, SABR was still associated with a worse OS (HR of 1.26; 95% CI: 1.10–1.45; *p =* 0.001). Median OS for the entire cohort was 43.6 months (95% CI 36.3–50.9): 53.0 months (95% CI 45.2–60.8 months) for thermal ablation versus 27.4 months (95% CI 22.1–32.7 months) for SABR (*p =* 0.0002) (Figure 3A). Overall survival at one, two, three and five years was 94%, 80%, 65% and 41% respectively for thermal ablation and 84%, 61%, 37% and 19% for SABR. Subgroup analyses revealed no heterogeneous treatment effects (Figure 4).

Two subgroup analyses, including (a) only treatment naïve patients and (b) only patients with CRLM < 3 cm, did not change the differential outcomes with a median OS of 50.3 months vs. 26.0 months (*p =* 0.0005) and 54.8 months vs. 30.7 months (*p =* 0.01) for thermal ablation versus SABR. 

Baseline variables associated with a worse DPFS in univariate analysis were female sex, higher age, higher comorbidity scores, node-positive disease, disease synchronicity, Fong’s clinical risk score and larger size of treated CRLMs. In both uni- and multivariate analysis there was no significant difference between thermal ablation and SABR with regards to the DPFS (HR = 1.07; 95% CI 0.93–1.22; *p =* 0.35) (Figure 3B).

### 3.4. Local Tumor Progression-Free Survival and Eventual Local Control

Baseline variables associated with a worse LTPFS were synchronous disease, high Fong’s clinical risk score, extrahepatic disease, higher number of CRLMs treated, larger size of treated CRLMs and treatment with SABR (Table 3). Median LTPFS was not reached in both groups. LTP was detected in 31 out of 144 patients (21.5%) and in 34 out of 400 CRLM (8.5%) in the thermal ablation group versus in 18 out of 55 patients (32.7%) and 20 out of 69 CRLM (29%) in the SABR group. In the thermal ablation group there were 45 CRLM >3 cm and LTP occurred in 16 of these CRLM, compared to 9/26 CRLM >3 cm in the SABR group. For patients with LTP, the median time to the detection of LTP per-tumor was 6.6 months (95% CI: 5.7–7.6) for thermal ablation and 9.0 months (95% CI: 7.5–10.5) for SABR. LTPFS per-tumor (HR = 1.35, 95% CI 1.11–1.65; *p =* 0.003) was worse in the SABR group and there was no significant difference between the SABR and thermal ablation groups in LTPFS per-patient (HR = 1.12, 95% CI 0.92–1.36; *p =* 0.278) (Figure 3C,D).

**Figure 5:**
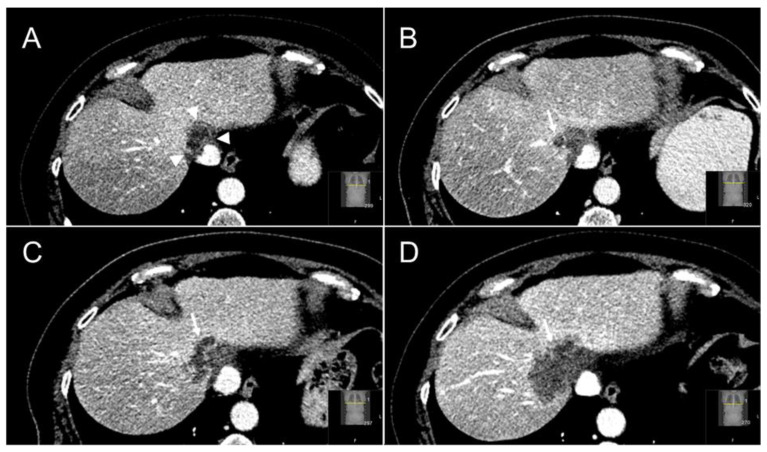
Follow-up cross-sectional ceCT images of a segment IVa-VIII CRLM treated with SABR. At three months ceCT showed a typical halo sign of hypoattenuation directly surrounding the irradiated, largely necrotic tumor (white arrowheads) and characteristic hyperattenuation of the perilesional liver parenchyma, which correlates with radiation induced hepatic fibrosis (**A**). At six months a solid ring-enhancing nodular lesion (white arrow) can be appreciated at the right-lateral margin of the irradiated tumor (**B**). This appearance is highly specific for the presence of local tumor progression post-SABR. Follow-up ceCT at six and 12 months (**C**,**D**) further confirmed the local tumor progression.

**Figure 6:**
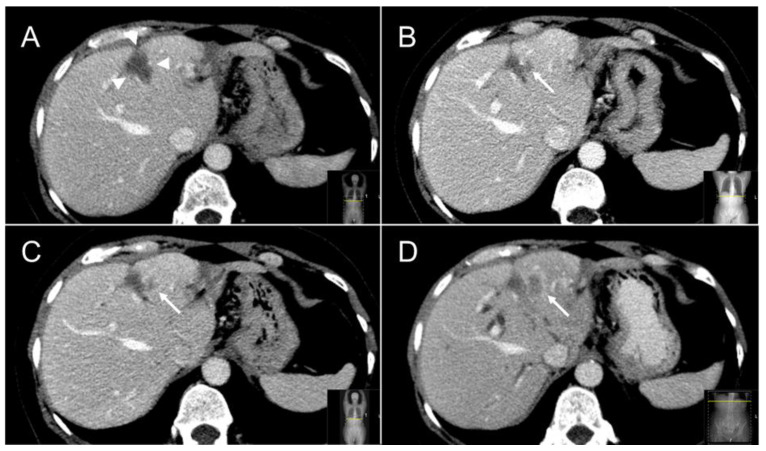
Follow-up cross-sectional ceCT images of a CRLM treated with thermal ablation. At six months a typical sharply demarcated hypoattenuating scar lesion (white arrowheads) is visible (**A**). At 12 months a characteristic “incomplete ring sign” (white arrow) at the left margin of the ablation zone can be appreciated (**B**), which, especially when combined with 18F-FDG avidity, is highly specific for local tumor progression following thermal ablation. Follow-up ceCT at 15 and 18 months (**C**,**D**) further confirmed local tumor progression.

The independent diagnostic radiologists’ re-evaluation (JHvW, IN) of suspected LTPs concurred in 80% (*n =* 16/20) of SABR treated LTPs and in all (*n =* 34/34) thermally ablated LTPs. The four discrepant cases were resolved in consensus between both diagnostic radiologists and the treating radiation oncologist (NH). For SABR the recurrences were described as solid and new or enlarging marginal lobulated incomplete-ring enhancing masses on ceCT in 14/20 LTPs (see Figure 5 for an example), as a new or augmenting marginal 18F-FDG PET avid mass abutting the irradiated CRLM in 5/20 LTPs and/or as a new or enlarging solid mass with diffusion restriction on ceMRI in 1/20 LTPs. For thermal ablation the detected recurrences were defined as new or enlarging, solid, marginal, lobulated incomplete-ring enhancing masses on contrast enhanced CT in 15/34 LTPs (see Figure 6 for an example), as new or enlarging marginal 18F-FDG PET avid mass abutting the ablated CRLM in 17/34 LTPs and/or as a new or enlarging solid mass with diffusion restriction on MRI in 2/34 LTPs.

**Figure 7:**
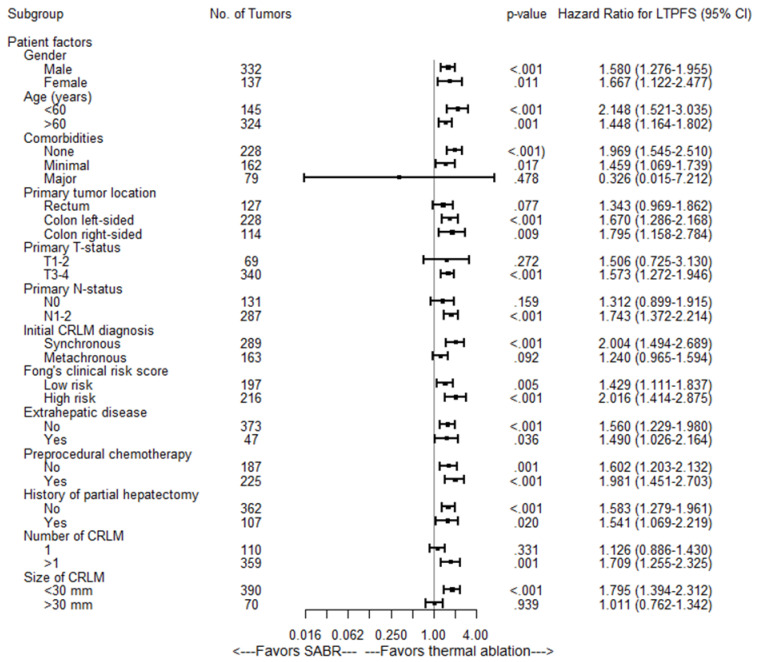
Univariate Subgroup Cox Regression Analyses of SABR versus thermal ablation associated with LTPFS per tumor.

Of 31 thermally ablated patients with LTP, 23 (23/34 tumors) were retreated using repeat thermal ablation (*n =* 20), partial hepatectomy (*n =* 1), IRE (*n =* 1) and SABR (*n =* 1). In 8 thermally ablated patients (11/34 tumors) local retreatment was deemed biologically futile due to widespread distant disease progression at first diagnosis of the LTP (competing risks). After a minimum follow-up of one year following the last local treatment, 15 patients (15/23 tumors) were alive and locally controlled. Three of the irradiated patients with LTP were locally retreated using partial hepatectomy (*n =* 1) and IRE (*n =* 2). In 15 irradiated patients (17/20 tumors) simultaneous detection of distant disease progression at first diagnosis of LTP prohibited retreatment (competing risks). After a minimum follow-up of one year following the last local treatment, two retreated patients (2/3 tumors) were alive and locally controlled. Local control per-tumor (HR = 1.90, 95% CI 1.44–2.50; *p <* 0.001) and per-patient (HR = 1.60, 95% CI 1.23–2.08; *p =* 0.001) was significantly worse in the SABR group (Figure 3E,F). Subgroup analyses revealed no heterogeneous treatment effects regarding LTPFS per-tumor (Figure 7).

**Figure 8:**
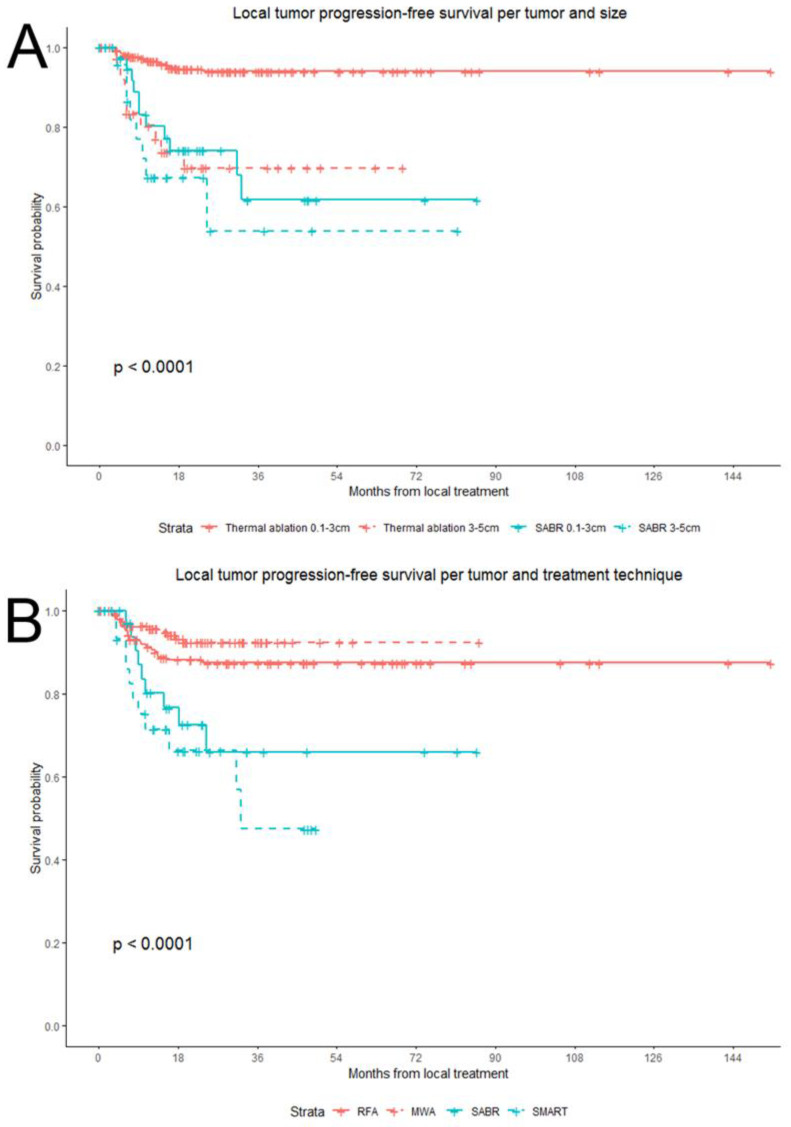
Local tumor progression-free survival curves (LTPFS) following thermal ablation (red) and stereotactic ablative radiotherapy (blue) for unresectable colorectal liver metastases according to subgroup analyses: stratified by CRLM < 3 cm versus 3–5 cm (**A**) and by specific treatment method radiofrequency ablation (RFA), microwave ablation (MWA), conventional stereotactic ablative radiotherapy (SABR) and selective MR-guided ablative radiotherapy (SMART) (**B**).

Log-rank test analysis of two subgroups, including (a) only treatment naïve patients and (b) only CRLMs <3 cm, did not change the outcome: thermal ablation resulted in a superior per-tumor LTPFS (*p <* 0.001). Thermal ablation of intermediate-size CRLM (3–5 cm) was associated with a significantly worse LTPFS (*p <* 0.001) when compared to small-size CRLM (<3 cm), whereas after SABR no significant differences were detected for intermediate-size versus small-size CRLM (*p =* 0.361), implying that SABR may be less susceptible to larger tumor sizes (Figure 8A). Univariate Cox regression showed that with regard to the per-tumor LTPFS, MWA was not superior to RFA (*p =* 0.167), and selective MR-guided ablative radiotherapy was not superior to conventional SABR (*p =* 0.317) (Figure 8B).

## 4. Discussion

For the local treatment of unresectable CRLM, in this study thermal ablation was superior to SABR with regard to OS, LTPFS per-tumor and eventual local control when including repeat treatments. Correcting for potential confounders and adding subgroup analyses for local treatment-naïve patients, for patients with only CRLM < 3 cm and for RFA versus MWA and conventional SABR versus MR-guided SABR, did not change overall outcomes. Though size of the CRLM was the strongest predictor for LTP in the thermal ablation group, and though size did not predict LTP in the SABR group, suggesting this technique to be less susceptible to changes in size, thermal ablation was still superior for tumors > 3 cm in size with regard to OS, DPFS, LTPFS and local control. The overall complication rate was low in both groups, with no 90-day mortality and no acute life-threatening complications. Though the total number of adverse events did not differ between the treatment groups, patients in the SABR group more often had lower grade events (CTCAE grade 1 or 2), whereas patients in the thermal ablation group more often experienced events requiring an intervention. The rate of serious but not life-threatening (CTCAE grade 3) adverse events was 0% (0/55 patients) for SABR versus 6.3% (9/144 patients) of patients treated with percutaneous thermal ablation. 

Two previous series, both retrospective cohorts, compared SABR to thermal ablation in patients with CRLM [22,23]. Stintzing and colleagues showed results comparable to the present series with a median OS of 52.3 months for thermal ablation vs. 34.4 months for SABR, though not reaching significance in their series of 60 patients (*p =* 0.06). In contrast to our results, the probability of LTP in their series was high, with hardly any CRLM eventually being locally controlled with either treatment method. Although Franzese and colleagues claim a superior local control for SABR, the justification is difficult to follow given the fact that 18.9% (*n =* 21 out 111 CRLM) of MWA treated CRLM recurred versus 19.4% (*n =* 20 out of 103 CRLM) in the SABR group and the attached freedom from local progression curve only shows 8 of these 20 events in the SABR group and 17 of the 21 events in the thermal ablation group because the displayed follow-up stops at 18 months. Differences in OS were not reported. Neither of these earlier series robustly define how to confirm LTP on cross-sectional imaging and neither describe eventual local control following repeat local treatments.

Several studies on SABR for both colorectal and non-colorectal liver metastases reported three-year local control rates of 48.3–91% and a three-year OS of 23–44% [24,25,26,27,28,29,30,31]. The largest series to date, a recent Dutch–Belgian registry covering 515 patients and 668 metastases, showed a three-year local control rate of 68% and three-year OS of 44% [31]. These outcomes are consistent with the present series, with a 64% three-year local control rate and a three-year OS of 35%. As thermal ablation is a repeatable technique, authors more often disclose the LTPFS following the first procedure and local control including repeat sessions whenever necessary. According to a recent meta-analysis that included 48 series, longer-term LTPFS ranges between 56–98% and eventual local control ranges between 86–100% [9]. Again our results reflect earlier series with a three-year LTPFS of 90% and a three-year local control of 97%.

The sensitivity and specificity of ceCT, 18F-FDG PET-CT and ceMRI to detect LTP following thermal ablation are well established, ranging between 83–92% and 95–100% respectively [14]. Though it can be difficult to assess routine cross-sectional imaging of liver tumors following SABR due to radiation-induced liver fibrosis and tissue reorganization within the irradiated zone [32,33], the accuracy in detecting LTP actually seems to compare well with follow-up after thermal ablation [32,33,34]. Jarraya and colleagues have described that the occurrence of a lobulated enhancement predicted LTP with a sensitivity of 89%, a specificity of 100% and an overall concordance rate of 97.9% [33].

Strengths of this comparative analysis are the relatively high number of patients and tumors assessed, the long-term follow-up, the baseline data accessed from a prospective registry and most importantly the multidisciplinary approach with image reassessments done by two abdominal radiologist referees, independently both from each other and from the treating physicians. Multivariate analysis with Cox regression could be used to reduce the impact of selection bias and several subgroup analyses were added to further reduce confounding and substantiate results.

Key limitations are the non-randomized nature and the unclearly defined criteria when to opt for thermal ablation versus SABR. Although anatomically the large majority of irradiated tumors would have also been eligible for thermal ablation and vice versa (the majority of thermally ablated tumors would have qualified for SABR), the exact reasons to opt for a certain treatment method are multifactorial and difficult to recollect by retrospectively searching the hospital’s electronic patient database. Another limitation is the long period of inclusion and its consequent gradual change in specific treatment methods, where for example MR-guided SABR is currently often favored over conventional SABR and where MWA seems to largely have replaced RFA. Though these limitations make our analysis at high risk of residual bias, the superior local effectiveness and repeatability of thermal ablation over SABR does show that the two techniques are not in equipoise. Further refinements to improve the efficacy of SABR seem necessary before randomized controlled trials can be considered for smaller tumors. However, given that differences in local effectiveness between the two treatment methods seem to shrink with increasing tumor size and given the excellent safety profile of SABR, the authors do believe a comparative study for tumors 3–5 cm in size is justified and warranted. Several ongoing randomized controlled trials will hopefully further elucidate the roles of thermal ablation and SABR to treat larger-size or difficult-to-reach CRLM (NCT04081168, NCT03654131 and NCT02820194).

To conclude, for the treatment of CRLM, in this study thermal ablation was superior to SABR with regard to OS, LTPFS and local control, albeit at the cost of a limited risk of serious adverse events. Future work, preferably in the form of a randomized clinical trial, should assess whether the worse outcomes following SABR were the effect of true differences in ablative treatment or merely a result of residual confounding.

## Figures and Tables

**Table 1:** Baseline characteristics of CRLM patients treated with thermal ablation or stereotactic ablative radiotherapy (SABR).

Factor		Total Cohort	Thermal Ablation	SABR	*p*-Value
		N	N	N	
Total		199	144	55	
Patient characteristics					
Sex					**0.010**
	Male	137 (68.8%)	107 (74.3%)	30 (54.5%)	
	Female	62 (31.2%)	37 (25.7%)	25 (45.5%)	
Age (years)	Median (IQR)	68.6 (15.9)	66.8 (16.5)	71.5 (13.0)	**0.006**
Charlson’s Comorbidity Index (CCI)					0.281
	Low CCI 0–5	90 (45.2%)	70 (48.6%)	20 (36.4%)	
	Moderate CCI 5–8	71 (35.7%)	49 (34%)	22 (40%)	
	High CCI >8	38 (19.1%)	25 (17.4%)	13 (23.6%)	
Location primary tumor					0.857
	Rectum	64 (32.2%)	45 (31.3%)	19 (34.5%)	
	Left-sided colon	93 (46.7%)	69 (47.9%)	24 (43.6%)	
	Right-sided colon	42 (21.1%)	30 (20.8%)	12 (21.8%)	
Primary T-status					0.813
	T1-2	27 (13.6%)	21 (16.4%)	6 (13%)	
	T3-4	147 (73.9%)	107 (83.6%)	40 (87%)	
	Unknown	25 (12.6%)	16	9	
Primary N-status					0.605
	N0	70 (35.2%)	53 (40.8%)	17 (35.4%)	
	N1-2	108 (54.3%)	77 (59.2%)	31 (64.6%)	
	Unknown	21 (10.6%)	14	7	
Primary M-status					**0.005**
	synchronous	94 (47.2%)	78 (56.9%)	16 (32.7%)	
	metachronous	92 (46.2%)	59 (43.1%)	33 (67.3%)	
	Unknown	13 (6.5%)	7	6	
Fong’s clinical risk score					**0.030**
	Low risk	110 (55.3%)	73 (59.8%)	37 (78.8%)	
	High risk	59 (29.6%)	49 (40.2%)	10 (21.3%)	
					
Extrahepatic disease at diagnosis					**0.004**
	No	147 (73.9%)	112 (90.3%)	35 (71.4%)	
	Yes	26 (13.1%)	12 (9.7%)	14 (28.6%)	
History of preprocedural chemotherapy					**0.083**
	No	109 (54.8%)	74 (58.7%)	35 (72.9%)	
	Yes	66 (33.2%)	53 (41.7%)	13 (27.1%)	
History of partial hepatectomy					0.174
	No	137 (68.8%)	95 (66%)	42 (76.4%)	
	Yes	62 (31.2%)	49 (34%)	13 (23.6%)	
Number of CRLM treated within procedure					
	Median	2 (1–18)	2 (1–18)	1 (1–5)	**0.0004**
Diameter of CRLM at procedure (mm)					**<0.0005**
	Median	21 (2–68)	14 (2–65)	29 (6–68)	

Note: IQR = Interquartile range, *p*-values in bold are *p*-values ≤ 0.1.

**Table 2:** Variables associated with OS in univariate Cox regression analysis and potential confounders in multivariate Cox regression analysis for OS when comparing SABR to thermal ablation (bold type significant confounders).

Univariate Analysis OS			Multivariate Analysis OS	
Variable	Hazard Ratio (95% CI)	*p* Value	Hazard Ratio	*p* Value
Treatment				
Thermal ablation	1 [reference]	**<0.001**	1 [reference]	**0.001**
SABR	1.29 (1.12–1.49)		1.27 (1.10–1.46)	
Sex				
Male	1 [reference]	0.374		
Female	0.83 (0.53–1.29)			
				
Age in years				
<68 years	1 [reference]	0.150		
>68 years	1.35 (0.90–2.01)			
Comorbidities				
None	1 [reference]	**0.034**	1 [reference]	0.204
Minor	1.08 (0.68–1.70)		0.98 (0.62–1.56)	
Major	1.92 (1.16–3.17)		1.54 (0.90–2.64)	
Location primary tumor				
Rectal	1 [reference]	0.282		
Left sided colon	0.89 (0.57–1.39)			
Right sided colon	1.36 (0.80–2.32)			
Primary T category				
T1-2	1 [reference]	0.469		
T3-4	1.28 (0.66–2.48)			
Primary N category				
N0	1 [reference]	**0.077**	1 [reference]	0.068
N1-2	1.51 (0.96–2.38)		1.54 (0.97–2.46)	
Timing of CRLM				
Metachronous	1 [reference]	0.722		
Synchronous	1.09 (0.72–1.64)			
Fong CRS				
Low risk	1 [reference]	0.180		
High risk	1.38 (0.88–2.16)			
Extrahepatic disease at diagnosis				
No	1 [reference]	0.844		
Yes	0.93 (0.51–1.73)			
Preprocedural chemotherapy				
No	1 [reference]	0.315		
Yes	0.80 (0.52–1.25)			
History of liver resection				
No	1 [reference]	**0.064**	1 [reference]	0.193
Yes	0.64 (0.41–1.02)		0.73 (0.45–1.17)	
Number of CRLM during procedure				
1 CRLM	1 [reference]	0.541		
>1 CRLM	0.892 (0.60–1.33)			
Diameter CRLM at procedure				
≤3 cm	1 [reference]	**0.004**	1 [reference]	**0.014**
3 cm	1.81 (1.21–2.70)		1.67 (1.11–2.51)	

Note: *p*-values in bold are *p*-values ≤ 0.1.

**Table 3:** Variables associated with LTPFS per-tumor in univariate Cox regression analysis and potential confounders in multivariate Cox regression analysis for LTPFS when comparing SABR to thermal ablation (bold type significant confounders).

Univariate Analysis LTPFS			Multivariate Analysis LTPFS	
	Per-Tumor			
**Variable**	**Hazard Ratio (95% CI)**	***p*** **Value**	**Hazard Ratio (95% CI)**	***p*** **Value**
Treatment				
Thermal ablation	1 [reference]	**<0.001**	1 [reference]	**0.003**
SABR	1.58 (1.31–1.90)		1.35 (1.11–1.65)	
Sex				
Male	1 [reference]	0.398		
Female	0.75 (0.39–1.46)			
Age in years				
<68.6 years	1 [reference]	0.363		
>68.6 years	1.28 (0,75–2.19)			
Comorbidities				
None	1 [reference]	0.331		
Minor	0.78 (0.44–1.39)			
Major	0.51 (0.20–1.31)			
Location primary tumor				
Rectal	1 [reference]	0.496		
Left-sided colon	0.95 (0.53–1.73)			
Right-sided colon	0.63 (0.28–1.41)			
Primary T category				
T1-2	1 [reference]	0.220		
T3-4	1.79 (0.71–4.56)			
Primary N category				
N0	1 [reference]	0.914		
N1-2	0.97 (0.53–1.78)			
Timing of CRLM				
Metachronous	1 [reference]	**<0.001**	1 [reference]	0.103
Synchronous	0.36 (0.21–0.63)		0.61 (0.34–1.11)	
Fong CRS				
Low risk	1 [reference]	**0.019**	1 [reference]	0.141
High risk	0.46 (0.25–0.88)		0.61 (0.32–1.18)	
Extrahepatic disease at diagnosis				
No	1 [reference]	**0.002**	1 [reference]	0.137
Yes	2.80 (1.48–5.30)		1.67 (0.85–3.29)	
Preprocedural chemotherapy				
No	1 [reference]	0.234		
Yes	0.69 (0.37–1.27)			
History of liver resection				
No	1 [reference]	0.576		
Yes	1.19 (0.65–2.19)			
Diameter CRLM at procedure				
≤3 cm	1 [reference]	**<0.001**	1 [reference]	**<0.001**
>3 cm	6.30 (3.67–10.83)		4.73 (2.65–8.45)	

Note: *p*-values in bold are *p*-values ≤ 0.1.

## Data Availability

The data presented in this study are available on request from the corresponding author.
